# Nonvolatile Modulation of Bi_2_O_2_Se/Pb(Zr,Ti)O_3_ Heteroepitaxy

**DOI:** 10.1021/acsami.4c02525

**Published:** 2024-05-15

**Authors:** Yong-Jyun Wang, Zi-Liang Yang, Jia-Wei Chen, Ruixue Zhu, Shang-Hsien Hsieh, Sen-Hao Chang, Hong-Yuan Lin, Chun-Liang Lin, Yi-Chun Chen, Chia-Hao Chen, Bo-Chao Huang, Ya-Ping Chiu, Chao-Hui Yeh, Peng Gao, Po-Wen Chiu, Yi-Cheng Chen, Ying-Hao Chu

**Affiliations:** † Department of Materials Science and Engineering, National Tsing Hua University, Hsinchu 300044, Taiwan; ‡ Graduate School of Advanced Technology, National Taiwan University, Taipei 106319, Taiwan; § Department of Materials Science and Engineering, National Yang Ming Chiao Tung University, Hsinchu 300093, Taiwan; ∥ International Center for Quantum Materials, School of Physics, Peking University, Beijing 100871, China; ⊥ Electron Microscopy Laboratory, School of Physics, Peking University, Beijing 100871, China; # National Synchrotron Radiation Research Center, Hsinchu 300092, Taiwan; ∇ Department of Electrophysics, National Yang Ming Chiao Tung University, Hsinchu 300093, Taiwan; ○ Department of Physics, National Cheng Kung University, Tainan 701401, Taiwan; ◆ Department of Physics, National Taiwan University, Taipei 106319, Taiwan; ¶ Department of Electrical Engineering, National Tsing Hua University, Hsinchu 300044, Taiwan

**Keywords:** nonvolatile modulation, Bi_2_O_2_Se, 2D semiconductor, ferroelectric, electronic potential

## Abstract

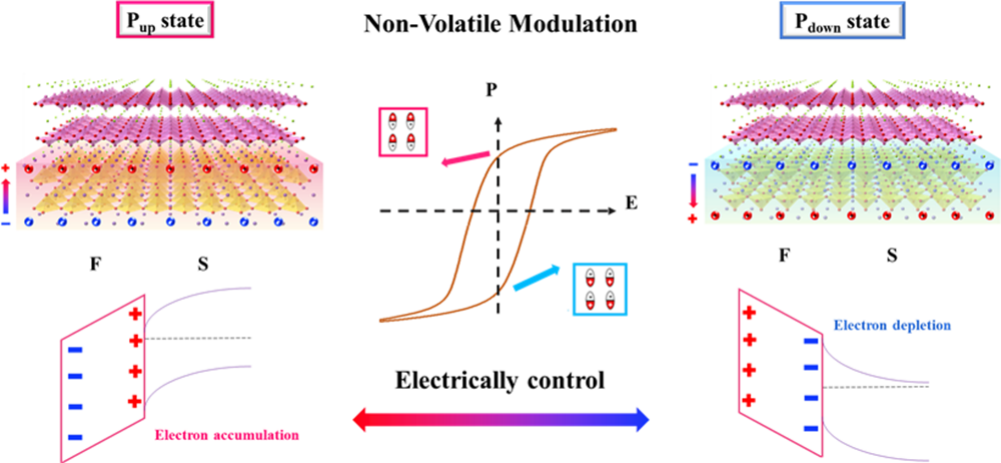

The pursuit of high-performance electronic devices has driven the research focus toward 2D semiconductors with high electron mobility and suitable band gaps. Previous studies have demonstrated that quasi-2D Bi_2_O_2_Se (BOSe) has remarkable physical properties and is a promising candidate for further exploration. Building upon this foundation, the present work introduces a novel concept for achieving nonvolatile and reversible control of BOSe’s electronic properties. The approach involves the epitaxial integration of a ferroelectric PbZr_0.2_Ti_0.8_O_3_ (PZT) layer to modify BOSe’s band alignment. Within the BOSe/PZT heteroepitaxy, through two opposite ferroelectric polarization states of the PZT layer, we can tune the Fermi level in the BOSe layer. Consequently, this controlled modulation of the electronic structure provides a pathway to manipulate the electrical properties of the BOSe layer and the corresponding devices.

## Introduction

Silicon has dominated the semiconductor industry since the invention of integral circuits (IC) in the late 1950s. However, with the advanced technology, the rapid development of the semiconductor industry follows the expectation based on Moore’s law, suggesting the upcoming subnanometer era. Thus, new materials exploration is crucial for encountering the challenges of scaling limitations and developing next-generation electronics. High-mobility semiconductors form the basis of modern electronics, leading to the scalable fabrication of high-performance devices. To fulfill such requirements, new 2D materials with high electron mobility, a sizable bandgap (∼1.2 eV), and excellent adaptability at the heterointerfaces are the top priority of fundamental research. In search of 2D materials, Bi_2_O_2_Se (BOSe),^1−5^ a quasi-2D material with various astonishing physical properties, is a good candidate for further investigation. Among various 2D materials, BOSe has attracted significant attention due to its unique properties. The most attractive feature is the existence of the native high-k oxide layer, Bi_2_O_5_Se, which can be obtained through thermal oxidation after BOSe growth. This behavior is similar to that of the widely used Si/SiO_2_.^6−8^ Moreover, the field-effect mobility based on the BOSe-related transistors can reach an ultrahigh value (>20,000 cm^2^/V·s). Besides, the abundant physical properties of BOSe also benefit various studies, such as optoelectronics,^9−11^ thermoelectric,^12−14^ memory,^15−17^ and some IoT applications.^18,19^ With these advantages, it is evident that BOSe has excellent potential to play a vital role in next-generation electronic technology.

After realizing the BOSe’s great potential, electronic modulation is crucial for device applications. The control of conduction and electronic structure has been achieved via the electric field, photoelectric effect, etc. However, expanding new control concepts to gain nonvolatile and reversible capabilities is essential. Several possible mechanisms can be utilized based on the electrostatic effects of either induced ferroelectricity or surface charge. In recent years, the increase of artificial intelligence (AI) applications has catalyzed the evolution of computing paradigms toward edge computing architectures. Within this landscape, ferroelectric transistors emerge as critical components, particularly in addressing the crucial aspect of nonvolatile memory. By controlling the intrinsic polarization properties of ferroelectric materials, FeFETs enable data retention, even in the absence of power, further reducing the inherent volatility associated with traditional memory architectures. Previous research has reported that ferroelectric polarization can effectively modify intriguing physical properties. Nonvolatile modulation on 2D semiconductor materials (no matter n or p type) can be achieved through ferroelectric polarization. Moreover, even the polarity of an ambipolar system, such as WSe_2_, can be determined by ferroelectric modulation. Chen et al.^20^ have demonstrated that utilizing two polarization directions of BiFeO_3_ to achieve p- and n-type WSe_2_, forming a gate-free p-n diode; Grezes et al.^21^ have reported that the appearance of two-dimensional electron gases (2DEGs) at oxide interfaces provides a highly efficient spin-to-charge current interconversion. With these understandings, nonvolatile ferroelectric polarization is believed to be a powerful concept that can significantly impact the material’s properties.

Several previous research studies have demonstrated that BOSe exhibits ferroelectricity at room temperature.^17,22,23^ The various indications, such as PFM images, transfer characteristics, and symmetry identification, imply that the existence of ferroelectricity in the BOSe itself might appear under certain conditions. However, the influence of ferroelectric materials on BOSe is not yet reported. In this work, we demonstrate a BOSe/PbZr_0.2_Ti_0.8_O_3_ (PZT)/SrRuO_3_ (SRO)/SrTiO_3_ heteroepitaxy to show the possibility for the integration of BOSe with complex oxides. Among these, the versatility of the perovskite family has great potential to trigger many intriguing physical properties.^24−26^ Perovskite oxides, including SrTiO_3_, LaAlO_3_, and (La,Sr)(Al,Ta)O_3_, share a similar 4-fold symmetry and exhibit excellent lattice matching with BOSe. Previous studies^27−33^ have reported that the growth of BOSe on the perovskite oxide substrates presents excellent heteroepitaxy. With this understanding, integration of BOSe with the complex-oxide system is expected. A ferroelectric PZT layer is added beneath BOSe to provide a strong polarization to alter band structure to gain nonvolatile and reversible capabilities. It is noticeable that PZT (*a* = *b* = 3.954 Å, *c* = 4.089 Å, tetragonal) is selected due to the structural compatibility with BOSe (*a* = *b* =3.891 Å, *c* = 12.213 Å, tetragonal), beneficial for obtaining a superior heteroepitaxy. Specifically, the Fermi level position in the BOSe layer could be modulated according to ferroelectric polarization. Two distinct polarized states (*P*_up_ and *P*_down_ states) are expected to offer the built-in electric fields, further altering the band structure and the electrical properties of BOSe. The combination of BOSe and ferroelectric PZT can be demonstrated in FeFET application. The ultrahigh field-effect mobility is expected to enable fast data computing, further improving the performance of related applications. In conclusion, the idea for tuning the electronic properties of BOSe via a nonvolatile and reversible concept is provided, delivering an avenue for modifying the novel material system.

## Result and Discussion

### Structural Characteristics of the Epitaxial BOSe/PZT/SRO/STO Heterostructure

**Figure 1:**
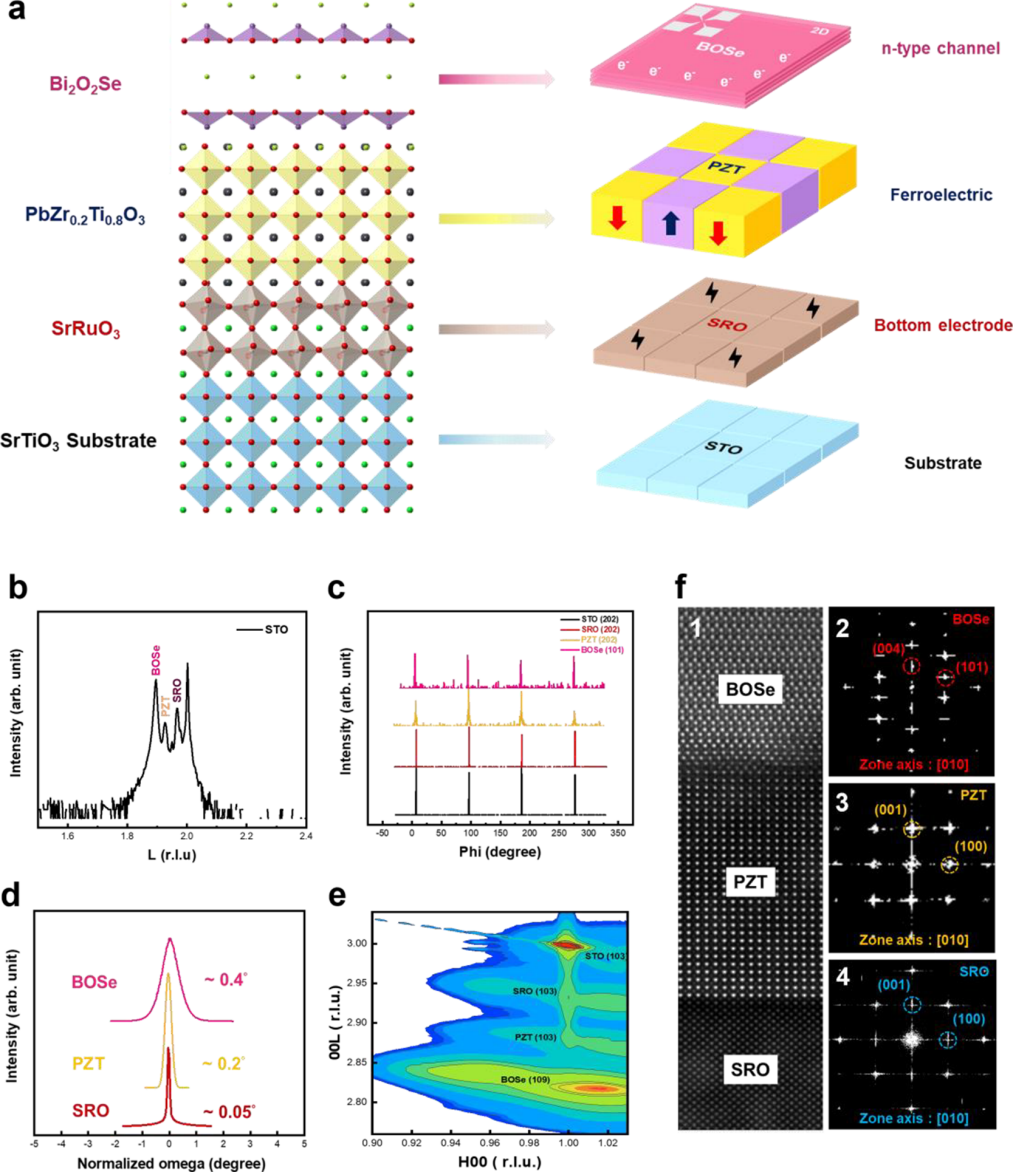
(a) Schematic diagram of the BOSe/PZT/SRO/STO heterostructure. (b) Normal scan of the (00L) series of STO with BOSe (006), PZT (002), and SRO (002) of the heterostructure. (c) Phi scans of STO(202), SRO(202), PZT(202), and BOSe(101). (d) Rocking curves of the as-grown SRO, PZT, and BOSe thin films. (e) Reciprocal space mappings of SRO, PZT, and BOSe on the STO substrate. (f) The cross-sectional STEM-HAADF images of the BOSe/PZT/SRO interfaces and their FFT patterns.

First, the structural characteristics of the BOSe/PZT/SRO/STO heterostructure were identified by X-ray diffraction (XRD). Figure 1a displays the schematic diagram of the BOSe/PZT/SRO/STO heterostructure grown by the pulsed laser deposition (PLD) method.^34^ The compatible lattices promote the heterointerface quality during the film stacking and are helpful for the subsequent measurements. To realize the crystalline orientation of the heterostructure, the XRD theta-2 theta scan shown in Figure 1b presents the pristine phase of BOSe, PZT, SRO, and STO (002) substrate. Only the (00L) series signals of BOSe, PZT, and SRO appear along with STO (00L) signals. To further verify it, the phi scans in Figure 1c are used to determine the in-plane (IP) orientation. The 4-fold symmetry along the (001) orientation is observed, and four sets of peaks at 90° intervals are displayed. The feature of aligned peaks leads to the epitaxial relationship of the heterostructure as (002)BOSe//(001)PZT//(001)SRO//(001)STO. These films show the same symmetry with the substrate, confirming superior heteroepitaxy. Moreover, the heterostructure performance strongly depends on the crystal quality. Thus, rocking curve measurements were conducted. The full width at half-maximum (fwhm) of SRO(002), PZT(002), and BOSe (006) peaks are ∼0.05, ∼ 0.2, and ∼0.4°, respectively, as shown in Figure 1d, proving the superior crystal quality of the heterostructure. After the fundamental understanding of the structure is realized, the next objective is to acquire the lattice strain of SRO, PZT, and BOSe films on the STO substrate. The reciprocal space mappings (RSMs) were carried out at room temperature, as shown in Figure 1e. The result suggests 0.6% in-plane compressive strain and 1.5% out-of-plane tensile strain for the SRO layer, 0.56% in-plane tensile strain and 1.5% out-of-plane compressive strain for the PZT layer; 1% in-plane compressive strain and 2.1% out-of-plane tensile strain for the BOSe layer. Such results show that the thin films were clamped to the STO substrate. Furthermore, the microstructure of the whole heterostructure was investigated by scanning transmission electron microscopy (STEM). The multilayer structure with the aid of the EDS mapping can be seen in the low-magnification images in Figure S1. To confirm the epitaxial characteristics, the cross-sectional STEM images taken along [100] _STO_ zone axis present the BOSe/PZT/SRO interfaces, as shown in Figure 1f, respectively. The sharp interfaces can be seen, and the reciprocal lattices in the fast Fourier transform (FFT) patterns of BOSe, PZT, SRO, and STO in the insets are indexed. These efforts have established the correctness of phases and epitaxial relationships, identifying the crystalline features.

### Electronic Potential Modulation by Ferroelectric Polarization

**Figure 2:**
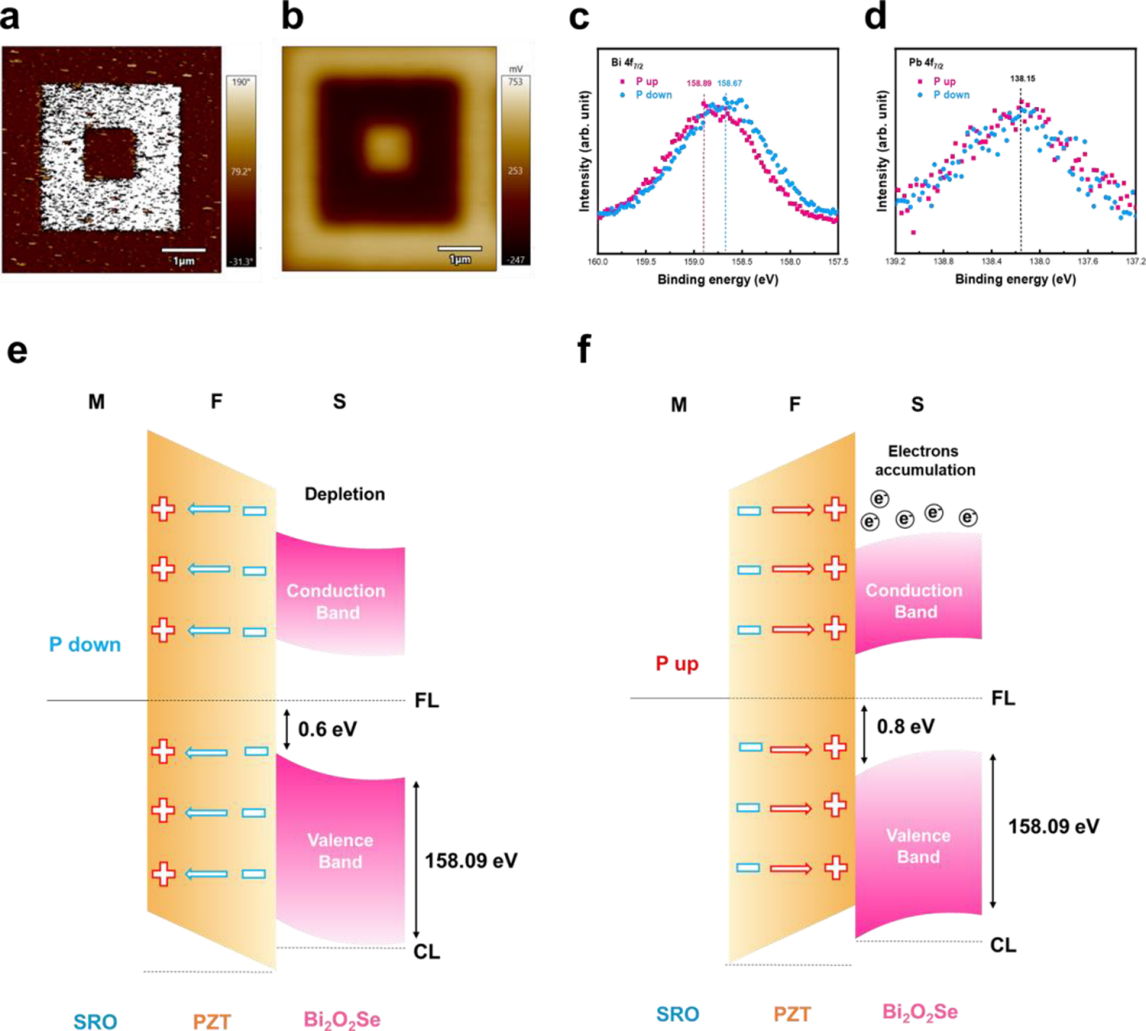
(a) PFM out-of-plane phase image after the poling process. (b) KPFM surface potential detected directly after the PFM measurement. The band structure of the BOSe/PZT heterointerface probed by XPS measurements. (c, d) BE alteration of Bi and Pb ions and (e, f) schematic diagrams illustrating the energy band alignment at the BOSe/PZT heterointerface under the *P*_down_ and *P*_up_ states.

Since the feature of crystal structure has been confirmed after a series of structural analyses, attention is paid to the electronic potential modulated by ferroelectric polarization. The two polarized states (*P*_up_ and *P*_down_) of the PZT layer offer permanent electric fields due to the nonvolatile behavior of ferroelectrics. Thus, the interface charge distribution is then modified, invoking the change in the band offset in the BOSe/PZT heterointerface. To further verify the change of local polarization, piezoresponse force microscopy (PFM) and Kelvin probe force microscopy (KPFM) were used for characterization. The out-of-plane polarization switching pattern (double square) was conducted by scanning conductive tips to investigate local polarization. The initial poling was carried out by applying a −8 V sample bias on a 5 μm by 5 μm area, followed by using an +8 V sample bias on a concentric 3 μm by 3 μm area, and followed by applying a −8 V sample bias on a concentric 1 μm by 1 μm area enclosed within the previous one. The topography and amplitude signal were extracted simultaneously and are shown in Supporting Information. After the poling process, the local reversal of the PZT polarization direction could be detected by the out-of-plane phase contrast shown in Figure 2a (The topography of the sample is presented in Figure S2a). Subsequently, the KPFM result measured right after the PFM experiment is presented in Figure 2b. Furthermore, the line scan is based on the KPFM images (Figure S2b,c) and shows a potential change of ∼0.2 eV between the *P*_up_ and *P*_down_ areas. Such a result reflects the injected electron attracted by positive-bound charges in the area of PZT upward polarization. Also, the potential difference in KPFM reveals spatially that the carrier concentration alters between two states.

A clear depiction of the specific changes in the band structure is essential to gain a deeper insight. The band structure modulation can be identified through high-resolution X-ray photoelectron spectroscopy (XPS) with spatial resolution at the National Synchrotron Radiation Research Center (NSRRC). Before measurements, two areas with 60 μm by 60 μm were defined as the *P*_up_ and *P*_down_ regions, supported by the DLP Maskless Exposure System and PFM poling process. The poling was done by applying a + 8 V tip bias inside one region, while the other was poled by using an −8 V tip bias. With these efforts, the beam spot of the XPS measurement can aim at the *P*_up_, *P*_down_, and the nonpoled regions. The binding energies (BE) of Bi ions in the top BOSe semiconductor layer and Pb ions in the ferroelectric PZT layer were measured, as shown in Figure 2c,d (XPS spectra are shown in Figure S3). After the alignment of the Pb 4f_2/7_ binding energy (138.15 eV) in the spectra, the core-level electron (CL) of Bi 4f_2/7_ shows peaks at 158.89 and 158.67 eV in the configurations of up- and down-polarized PZT layer, respectively. Meanwhile, with the results of the valence band maximum scans in Figure S3h,i, the schematic diagram illustrating the band alignment at the BOSe/PZT heterojunction is shown in Figure 2e,f, representing the *P*_up_ and *P*_down_ states. For the n-type BOSe semiconductor layer, the increase of Bi 4f_2/7_ binding energy caused by the *P*_up_ state indicates the increase of carrier concentration and results in higher conductivity, since the Fermi level is shifted closer to the conduction band. On the other hand, the decrease of Bi 4f_2/7_ binding energy detected in the *P*_down_ region suggests a lower carrier concentration. It leads to lower conductivity due to electron depletion. The depletion width can be obtained by the formula below:
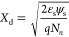
where *X*_d_ is the depletion width, ε_s_ is the dielectric constant of the semiconductor materials, ψ_s_ is the modulated electronic potential, *q* is the Coulomb’s constant, and *N*_n_ is the carrier concentration of the n-type semiconductor materials.

Based on the calculation from this formula, the depletion width was calculated as ∼5 nm, denoting the maximum space charge width. The polarization states of the PZT can induce the binding energy change in the BOSe layer at ∼0.2 eV, which is consistent with the potential difference extracted by the line scan analysis of KPFM results in the Supporting Information. This result delivers evidence of modulation of the BOSe semiconductor layer by ferroelectric polarization.

**Figure 3:**
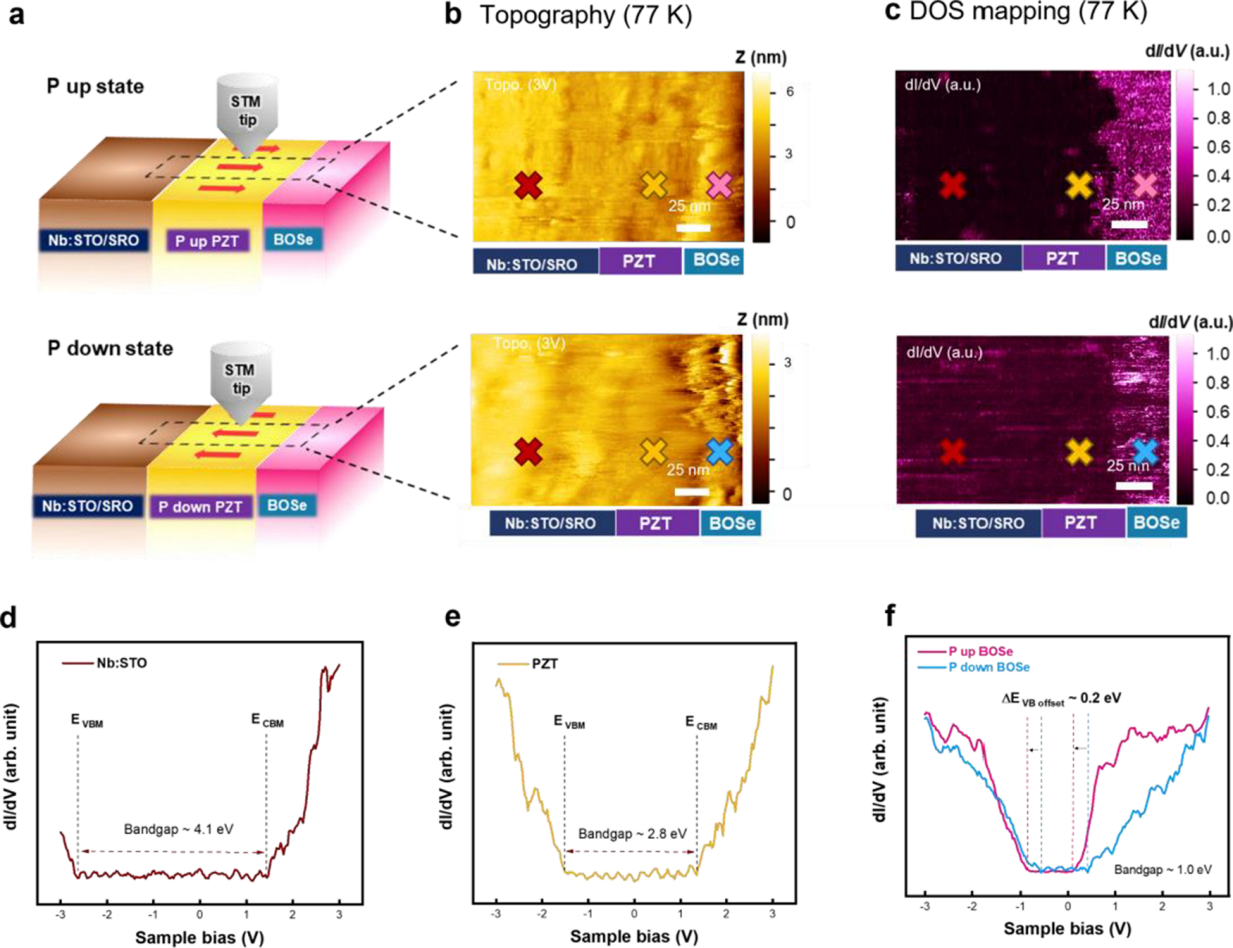
(a) Schematic diagram of the scanning tunneling microscopy with the comparison of the *P*_up_ and *P*_down_ states. (b, c) Topography and DOS mapping images from the cross-sectional direction at 77 K. The spatial spectroscopic measurements on (d, e) Nb:STO/SRO and PZT interfaces. (f) BOSe of BOSe/PZT/SRO/Nb:STO system under the *P*_up_ and *P*_down_ states.

To further reveal the PZT polarization effect on the BOSe electronic structure, cross-sectional scanning tunneling microscopy (XSTM) was employed to study the local electronic structure across the interfaces of the heterostructure. The geometry of the measurement is illustrated in Figure 3a. The XSTM topography and density of states (DOS) mapping images of the substrate/PZT/BOSe heterojunction were acquired in constant current mode, as shown in Figure 3b,c. The DOS mapping images indicate the electronic structure characteristics for each material at a certain energy level relative to the Fermi level. Although the topography image remains unchanged across different polarizations, the DOS of BOSe exhibits significant changes due to polarization, as shown in Figure 3c. Specifically, the color contrast in BOSe is more pronounced in the *P*_up_ condition, indicative of an increased DOS in BOSe triggered by *P*_up_ PZT. Once we differentiated the specific materials according to the DOS mappings and topography, we conducted the scanning tunneling spectroscopy (STS) measurements to investigate the electronic structure of each material, as shown in Figure 3d–f. The conduction band and valence band offset of BOSe with a bandgap of ∼1.0 eV is shifted downward about 0.2 eV when the polarization direction of the bottom PZT film changed from *P*_down_ to *P*_up_, implying the electron doping induced by substrate polarization. The increase in carrier concentration and the decrease in band offset caused by *P*_up_ PZT are consistent with the KPFM measurements.

### Investigation on the Macroscopic Electronic Properties

**Figure 4:**
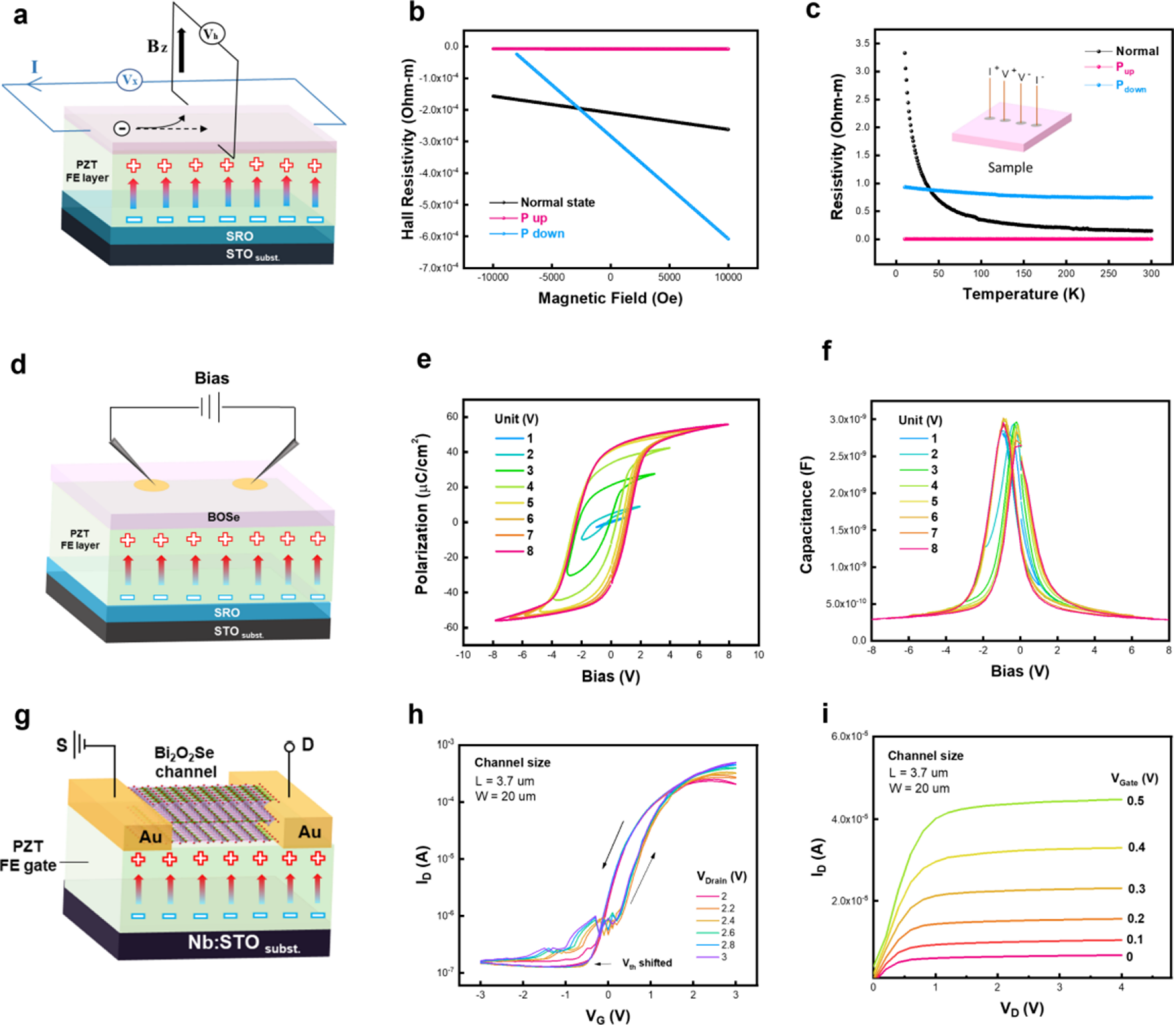
Electron characteristics of the synthesized FeFET system. (a) Schematic diagram of the Hall measurement. (b, c) Results of the Hall measurements and resistivity-temperature curves for BOSe under nonpoled, *P*_up_, and *P*_down_ states. (d) Schematic diagram of the *P*–*V* and *C*–*V* loop measurements on the MFS capacitor. (e, f) Results of the *P*–*V* and *C*–*V* loop measurements and increasing gate voltage from 1 to 8 V at an AC frequency of 10 kHz. (g) Schematic diagram of the fabricated bottom-gate FeFET device. (h, i) The transfer characteristics (*I*_D_–*V*_G_ and *I*_D_–*V*_D_) of the FeFET.

The insight into the modulated electronic potential has been observed from the vertical and lateral directions. Subsequently, the macroscopic electronic properties can provide more information to identify the interaction between BOSe and PZT. Here, three sets of measurements are presented to characterize the macroscopic electronic properties of this synthesized FeFET system. Due to the change in the interior electronic potential, the carrier concentration of the BOSe layer has been manipulated according to two distinct poling states. The schematic diagram of the measurement of electron transport properties is shown in Figure 4a. Before the measurements, the samples were polarized by a conductive tip with −8 and +8 V through a large-area poling system. With this effort, three conditions (normal, *P*_up_, and *P*_down_) were created to compare the influence of the different poling states on the BOSe layer. The results of the Hall measurements in Figure 4b show a noticeable change in the carrier concentration, according to two distinct poling conditions. The modified carrier concentration can be calculated according to the below formula:
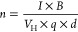
where *n* (#/cm^3^) is the carrier concentration, *q* is the Coulomb constant, *d* is the BOSe layer thickness, *V*_H_ is the applied voltage, *I* is the induced current, and *B* is the magnetic field. According to this formula, the absolute value of the fitting slope is inversely proportional to the carrier concentration. Based on the figure, the unpoled BOSe represents a black line, suggesting an n-type semiconductor feature due to the negative slope. Subsequently, the red line indicates a higher carrier concentration for the BOSe under the *P*_up_ state. Such an electron accumulation originates from the downward bending of the band structure. Furthermore, the blue line suggests a lower carrier concentration for the BOSe under the *P*_down_ state. The electron depletion happens when the band structure bends upward. On the other hand, the different poling conditions also affect the resistivity features. The relationship between the resistivity and temperature is shown in Figure 4c. According to the results, the unpoled BOSe (black line) presents a typical semiconductor feature and temperature. Meanwhile, the BOSe under the *P*_up_ (red line) and *P*_down_ (red line) states shows the conductor and insulator behaviors, respectively. Such results are consistent with the STS measurements (Figure S4). Based on the spectroscopy, the electronic behaviors of BOSe under three states show obvious differences, which can be seen as three materials with different band structures. The carrier concentration decides the resistivity features, which greatly corresponds to the results of electron transport properties and the investigation of the modified electronic potential.

Polarization–voltage (*P*–*V*) loop measurements were carried out on the synthesized FeFET system to study the behavior of the polarization characteristics. The presented architecture can be seen as a metal/ferroelectric/semiconductor (MFS) capacitor consisting of 500 nm PZT and 5 nm BOSe, as illustrated in Figure 4d. During the measurements, a 10 kHz triangular-wave voltage (*P*–*V* loops with different AC frequencies are shown in Figure S5a) was applied to the bottom electrode (SRO) side. Figure 4e shows the *P*–*V* characteristics of the fabricated MFS capacitor with applied bias from 1 to 8 V. In a ferroelectric capacitor, the coercive fields of the hysteresis *P*–*V* loop should be nearly the same in both directions. However, a significant shift can be usually seen in an MFS capacitor. This phenomenon originates from the electrostatic imbalance of the MFS heterostructure since one side of the ferroelectric capacitor is in touch with the semiconductor. Such a fact causes the change of the effective electric fields, leading to the shift of the *P*–*V* loops. The remanent polarization (2Pr) and coercive voltage (*V*_c_) in the synthesized MFS cap are shown in (Figure S6). On the other hand, the capacitance–voltage (*C*–*V*) loop measurements were also conducted to investigate the capacitance behaviors during the polarization switching. The *C*–*V* loops of the synthesized MFS capacitor are presented in Figure 4f with an applied bias from 1 to 8 V. Based on the results, the butterfly-like loops can also be observed when the applied bias is 8 V, suggesting the existence of ferroelectric behavior. These measurements offer an opportunity to capture the polarization switching behaviors in the ferroelectric gates at given device conditions, providing information toward the characterization of underlying device physics in FeFETs.

The interactions between the BOSe channel material and the ferroelectric PZT were fully verified according to the electron transport and polarization switching characteristics. Subsequently, the transfer characteristics of this fabricated FeFET system were studied through a device demonstration. The source and drain regions were created with a channel length of 5 um and a channel width of 20 um. Then, 30 nm Au/5 nm Ti were deposited by E-gun as the source and drain. The schematic of the fabricated bottom-gate FeFET device is shown in Figure 4g. In terms of the transfer characteristic measurements, the drain current (*I*_D_) was measured under a gate voltage (*V*_G_) from −1 to 3 V with increasing drain voltage (*V*_D_) from 2 to 4 V. As shown in Figure 4h, a four-order change on the ratio of the on/off current with the memory window of ∼0.4 V is observed, suggesting that the carriers in the BOSe channel can be controlled by the ferroelectric PZT gate effectively. A counterclockwise *I*_D_–*V*_G_ curve hysteresis indicates the ferroelectric conversion behavior from the PZT layer, in which the observed hysteresis regions in the *I*_D_–*V*_G_ loops originated from the quasi-static ferroelectric behavior^35,36^ (Figure S5) of the synthesized MFS capacitor. The calculated SS value based on the result is ∼4.1 × 10^7^. Compared to those common FETs, the more considerable SS value is attributed to the global bottom-gate layout. The voltage cannot significantly contribute to the gate control compared with the top-gate device layout. Moreover, for calculating the mobility of the FET, the *Y*-function is applied to extract the value of the mobility. The equation is listed below:
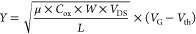

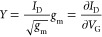
where *g*_m_ is the derivative of *I*_D_–*V*_G_ curves, μ is the mobility, *C*_ox_ is the capacitance of the gate oxide, *W* is the channel width, and *L* is the channel length. Based on the equation, a mobility of 1597.6 cm^2^/V·s can be obtained. The detailed calculation is shown in Supporting Information (Figure S7). Meanwhile, the retention and endurance data are presented in Figure S8, suggesting the robustness of the synthesized FeFET device. Furthermore, the *I*_D_–*V*_D_ curves were also measured under *V*_D_ from 0 to 4 V with increasing *V*_G_ from 0.1 to 0.5 V, as shown in Figure 4i. From the results, the operating regions and the threshold voltage under different conditions can be achieved. With this effort, the amplified and saturation regions can also be defined clearly. On the other hand, to verify that the contribution of BOSe’s ferroelectricity will not affect the results, the PZT ferroelectric gate has been exchanged for the paraelectric STO one. From the observation of Figure S9, the typical hysteresis window is hardly observed in the *I*_D_–*V*_G_ curves. Such a result further verifies the tiny influence of BOSe, and the effect of nonvolatile modulation is contributed from the ferroelectric PZT layer. Based on the macroscopic evidence, it can be certain that the on and off states of the BOSe channel can be significantly controlled by the ferroelectric PZT gate, offering a new control method to manipulate the electron behaviors of 2D BOSe.

## Conclusions

This work delivers an excellent idea to control the 2D BOSe channel material. A FeFET system consisting of an n-type channel material BOSe and a ferroelectric gate PZT is fabricated to obtain the nonvolatile modulation on the electronic structure. After a series of structural characterizations, the fabricated BOSe/PZT/SRO/STO (100) heterostructure presents single-crystal-like crystallinity. The modulation of the electronic characteristics through a nonvolatile method with the comparison of three distinct states (normal, *P*_up_, and *P*_down_) has been investigated. The polarized PZT layer can be seen as an external electric field in which the different polarization switching behaviors lead to the change of BOSe’s electronic characteristics. Through the deep exploration of the electronic structure, electron accumulation and depletion behaviors have been observed, further shedding light on the band structure, including the establishment of the band offset. Last, investigating the macroscopic electronic properties of the synthesized FeFET system provides another insight into the modulated electronic behaviors. The electron transport measurements show the changed carrier concentration and resistivity behaviors induced by ferroelectric polarization. Such a fact corresponds to the investigation of the modified electronic potential. Moreover, the polarization-switching behaviors of the synthesized MFS capacitor were also studied. Unlike a typical ferroelectric capacitor, the polarization switching during the voltage sweep leads to a change of carriers, further deforming the *P*–*V* characteristics. However, the control of the on and off states in the BOSe channel layer can be directly observed through the transfer characteristics of a FeFET device. With this effort, the control of the BOSe channel material by a ferroelectric gate as a transistor device has been demonstrated in this study. In summary, an idea for tuning the electronic characteristics of BOSe via a nonvolatile and reversible concept is delivered.

## Methods

### Sample Preparation

The BOSe/PZT/SRO/STO heterostructure was fabricated via PLD with commercial BOSe, PZT, and SRO targets. (SRO is the bottom electrode for electrical analysis). Commercial STO single crystals were used as the substrates. The vacuum chamber was evacuated to a pressure of 1 × 10^–6^ Torr before deposition. First, SRO was grown on the substrate at 680 °C under 100 mTorr of O_2_ pressure. Second, PZT was deposited under the same pressure and substrate temperature. Third, the BOSe layer was grown on the PZT layer at 405 °C under 100 mTorr of the O_2_ pressure. Lastly, the cooling process was conducted with a cooling rate of 0.3 °C/s.

### X-ray Diffraction

High-resolution X-ray diffraction techniques were performed to verify the crystal structures and epitaxial relationships of the synthesized heterostructure. L-scan, rocking curves, reciprocal space maps (RSMs), and phi-scans were recorded by using the light source in NSRRC.

### X-ray Photoelectron Spectroscopy

Before the measurements, two square windows with 60 × 60 μm^2^ surrounded by 50 nm Au were created, supported by the DLP Mask-less Exposure System. The polarization was carried out inside these two windows by applying a +8 V tip bias inside one window, while the other was polarized using an 8 V tip bias. Then, XPS measurements with a high spatial resolution were performed at the NSRRC in Hsinchu, Taiwan, at photon energies of 620 eV. All measurements were carried out at room temperature.

### Scanning Tunneling Spectroscopy

The PZT/BOSe samples were cleaved in an ultrahigh vacuum (UHV) STM chamber with a base pressure of ∼7 × 10^–11^ mbar at 20 K. Samples were then transferred to an STM scan head and measured at 77 K.

### Macroscopic Electron Characteristics

First, the Hall and resistivity measurements were carried out through a physical property measurement system. In the Hall measurement, the magnetic field was applied as 2 T, conducted at room temperature, while the resistivity–temperature curves were measured at decreasing temperatures from 300 to 10 K. Second, the polarization switching behaviors and MFS capacitance features were investigated under 100 mV AC voltage, 10 kHz AC frequency, and the voltage was measured from 1 to 8 V, measured by a commercial instrument for ferroelectric properties (TFAnalyzer3000, aixACCT Systems). Finally, the transfer characteristics were measured with a commercial B1500A system to obtain the performance of the synthesized FeFET device.

### Transmission Electron Microscopy

TEM samples were prepared by focused ion beam etching (Helios G4) etching. HAADF-STEM images and EDS were acquired using an aberration-corrected FEI Titan Themis G2 operating at 300 kV. The convergence semiangle for imaging is 30 mrad, and the collection semiangle range is 50–200 mrad.

## Data Availability

All the data needed to evaluate the conclusions in the paper are present in the paper and the Supporting Information.

## Supporting Information Available

Low-mag TEM image and the EDS mapping of the BOSe/PZT/SRO/STO heterostructure; AFM image for the sample topography; XPS spectra; schematic diagram; frequency-dependent P-E loops for the BOSe/PZT capacitor; coercive voltage; calculation of the field-effect mobility through Y-function calculation; retention and endurance data of the FeFTT; and schematic diagram of BOSe/STO/Nb:STO (PDF)
