# Supplementary material for: Nonvolatile Modulation of Bi2O2Se/Pb(Zr,Ti)O3 Heteroepitaxy
Source: ACS Appl Mater Interfaces. 2024 May 15;16(21):27523–31. doi: 10.1021/acsami.4c02525 (PMC11145581; doi:10.1021/acsami.4c02525)
Supplement: Supplementary file 1 — am4c02525_si_001.pdf [file am4c02525_si_001.pdf]

Supporting Information

Title: Non-Volatile Modulation of $Bi_2O_2Se/Pb(Zr,Ti)O_3$ Heteroepitaxy

Yong-Jyun Wang[1], Zi-Liang Yang[2], Jia-Wei Chen[3], Ruixue Zhu[4,5], Shang-Hsien Hsieh[6], Sen-Hao Chang[7], Hong-Yuan Lin[8], Chun-Liang Lin[7], Yi-Chun Chen[8], Chia-Hao Chen[6], Bo-Chao Huang[9], Ya-Ping Chiu[2,9], Chao-Hui Yeh[10], Peng Gao[4,5], Po-Wen Chiu[10], Yi-Cheng Chen[1*], and Ying-Hao Chu[1,3*]

Affiliation

[1]Department of Materials Science and Engineering, National Tsing Hua University, Hsinchu 300044, Taiwan

[2]Graduate School of Advanced Technology, National Taiwan University, Taipei 106319, Taiwan

[3]Department of Materials Science and Engineering, National Yang Ming Chiao Tung University, Hsinchu 300093, Taiwan

[4]International Center for Quantum Materials, School of Physics, Peking University, Beijing 100871, China

[5]Electron Microscopy Laboratory, School of Physics, Peking University, Beijing 100871, China

[6]National Synchrotron Radiation Research Center, Hsinchu 300092, Taiwan

[7]Department of Electrophysics, National Yang Ming Chiao Tung University, Hsinchu 300093, Taiwan

[8]Department of Physics, National Cheng Kung University, Tainan 701401, Taiwan

[9]Department of Physics, National Taiwan University, Taipei 106319, Taiwan

[10]Department of Electrical Engineering, National Tsing Hua University, Hsinchu 300044, Taiwan

*Correspondence to: yhchu@mx.nthu.edu.tw

EDS Mapping of the BOSe/PZT/SRO/STO heterostructure

The low-mag TEM cross-sectional image of the synthesized BOSe/PZT/SRO/STO heterostructures shown in Figure S1 allows us to observe the interfaces among layers. Moreover, the EDS mapping of the overall heterostructure presents the elemental distribution of each material. With these efforts, the correctness of the architecture can be confirmed.

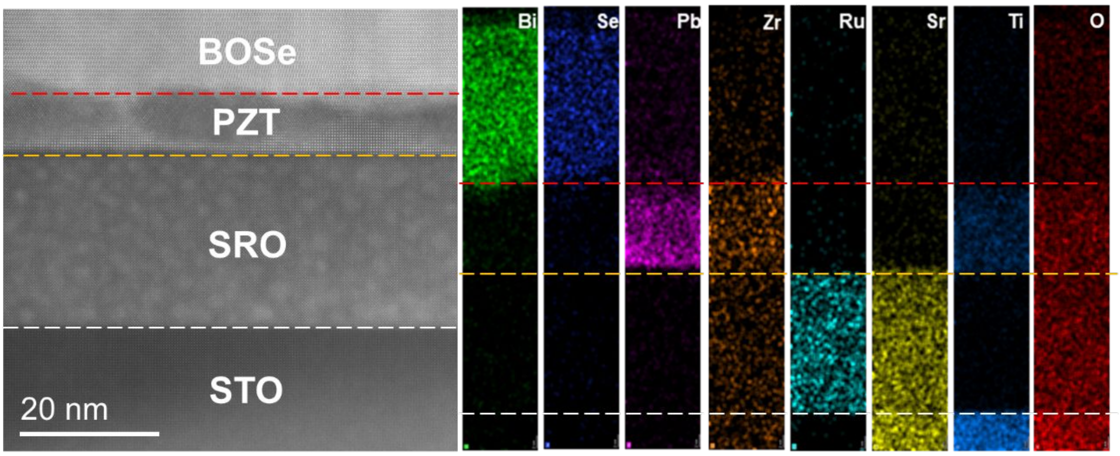

Figure S1. The low-mag TEM image and the EDS mapping of the BOSe/PZT/SRO/STO heterostructure.

SPM Results

Figure S2a shows the sample topography with no significant pinhole or crack in the sample. On the other hand, the KPFM image and the corresponding result of the line scan are presented in **Figure S2b,c**. The measurements were consequently conducted after the PFM measurement. From **Figure S2b,** a clear contrast with a double square shape can be observed, convincing the modulation of the surface potential via ferroelectric polarization. Consequently, the line scan of the KPFM image shows a modulation value of ~0.2 eV.

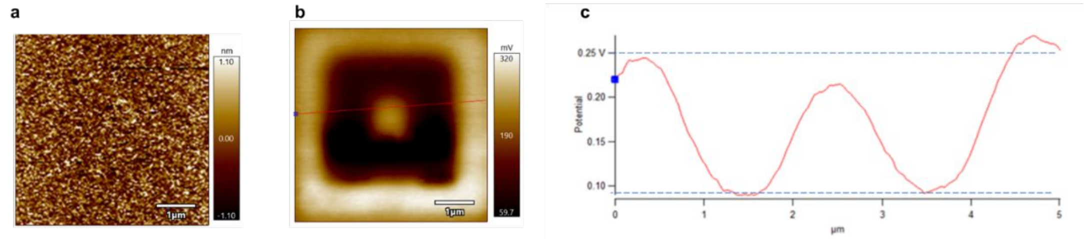

Figure. S2. a The AFM image for the sample topography. **b** KPFM image with **c** line scan, indicating the surface potential change for ~0.2 eV.

XPS Results

The XPS spectra for each element inside the BOSe/PZT heteroepitaxy with survey spectrum are shown in Figure S3 a-g. According to the results, each element's existence and valence state can be verified. Moreover, the results of the valence band maximum (VBM) scan for the BOSe and PZT layers are shown in Figure S3h and Figure S3i. The pointed intersections on the figures suggest the potential difference between the Fermi-level and VBM.

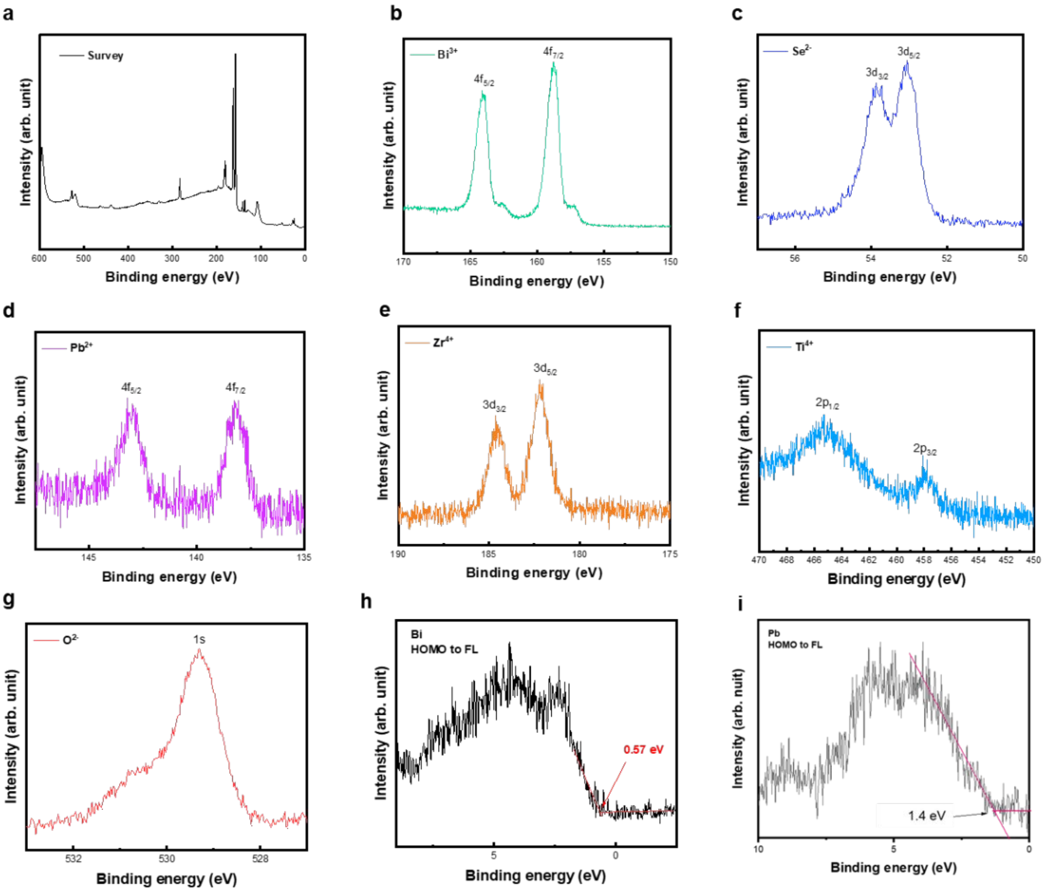

Figure. S3. The XPS spectra for **a** survey spectrum, **b** Bi 4f, **c** Se 3d, **d** O 1s, **e** Pb 4f, **f** Zr 3d, **g** Ti 2p, **h** BOSe VBM, and **i** PZT VBM.

The results of STS measurements

Figure S4 shows the results of the STS measurements for the samples under different polarized states. According to the spectroscopy, the BOSe electronic potential under three states shows obvious differences, which can be seen as three different band structures. Such results are consistent with the R-T and Hall measurements.

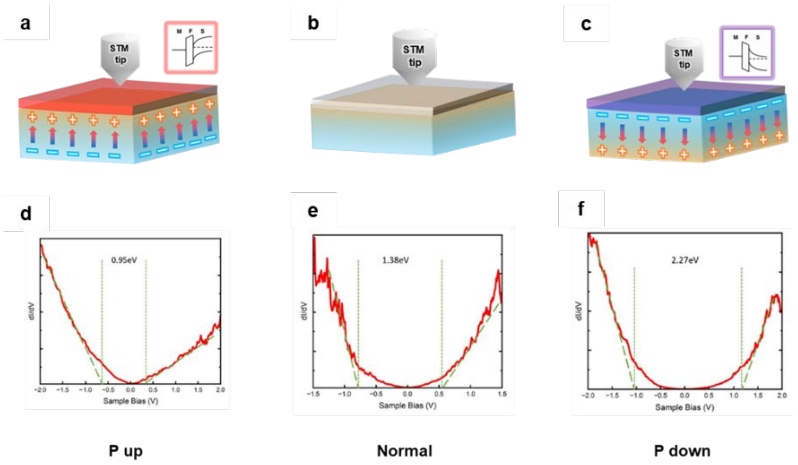

Figure. S4. The schematic diagram of **a** P up, **b** normal, and **c** P down states. The results of the STS measurements for **d** P up, **e** normal, and **f** P down states.

The quasi-static PZT polarization

The frequency-dependent PE loop measurements of the BOSe/PZT system along with the power-law fitting results. Based on the results, the coercive fields decrease along with the reduction in the AC frequency. Such results can be used to explain the smaller hysteresis window of the I_d-V_g curves.

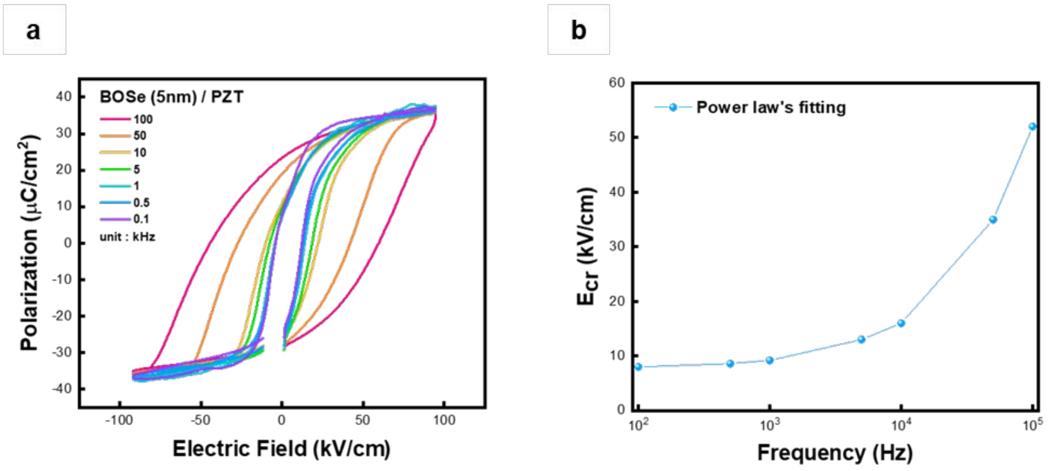

Figure. S5. a The frequency-dependent P-E loops for the BOSe/PZT capacitor. **b** The power law's fitting for the frequency-dependent P-E loops.

The coercive voltage (Vc) and remanent polarization (2Pr) of the synthesized MFS cap

For a MFS cap, the remanent polarization (2Pr) and coercive voltage (Vc) will not be symmetric for positive and negative bias. **Figure S5 a** shows the Vc of the synthesized MFS cap. From the results, an orientation of decreasing Vc for positive and negative bias can be observed, suggesting a n-type MFS cap feature. On the other hand, **Figure**

S6 b shows the 2Pr of the synthesized MFS cap along the direction of positive and negative bias. According to the figure, a non-symmetric value can be seen, which is consistent to the result in **Figure S6 a**.

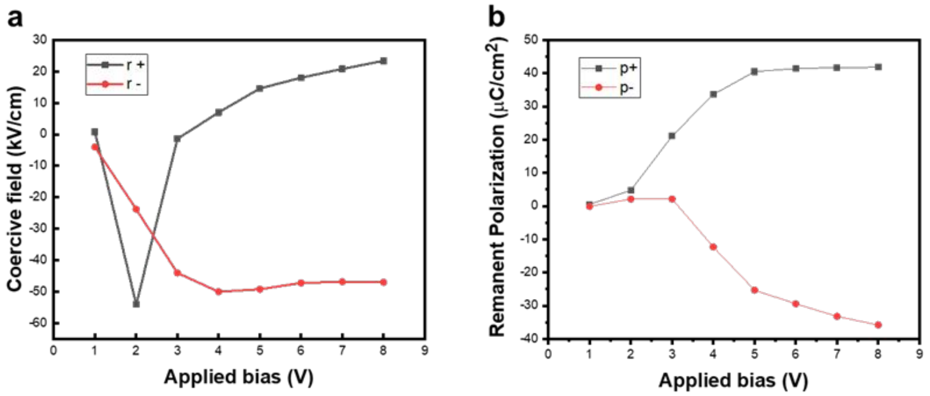

Figure S6. a The coercive voltage (V_c) and **b** The remanent polarization ($2P_r$) of the BOSe/PZT MFS capacitor.

Y-function calculation

$V_d = 2.6$ V, $V_{th} = 1$ V

$C_{ox} = 7.96E\text{-}6\ F/cm^2$, W = 20 μm, L = 3.7 μm

$$g_m = \frac{\partial I_D}{\partial V_G} \qquad Y = \frac{I_D}{\sqrt{g_m}}$$

$$Y = \sqrt{\frac{\mu * C_{ox} * W * V_{DS}}{L}} * (V_G - V_{th})$$

$$\frac{Y}{(V_G - V_{th})} = \sqrt{\frac{\mu * C_{ox} * W * V_{DS}}{L}} = \text{slope}$$

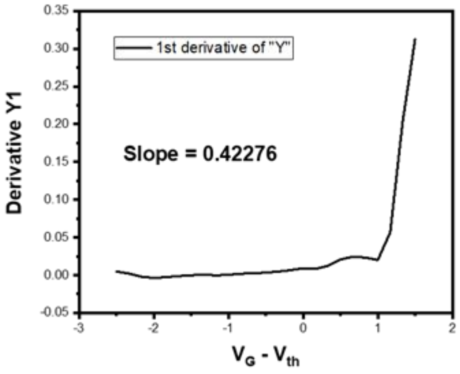

$$slope = 0.42276 = \sqrt{\frac{\mu * 7.96E - 6 * 20 * 2.6}{3.7}} \qquad \mu = \frac{0.42276^2 * 3.7}{7.96E - 6 * 2.6} * \frac{3.7}{20} = 1597.6\ cm^2/V*s$$

Figure. S7. The calculation of the field-effect mobility through Y-function calculation.

The endurance and retention data

To identify the stability of the fabricated FeFET device, the retention and endurance measurements were conducted and shown in **Figure S7**. From the results, the transfer characteristics are still robust even after 100-hour and 1000-cycle measurements.

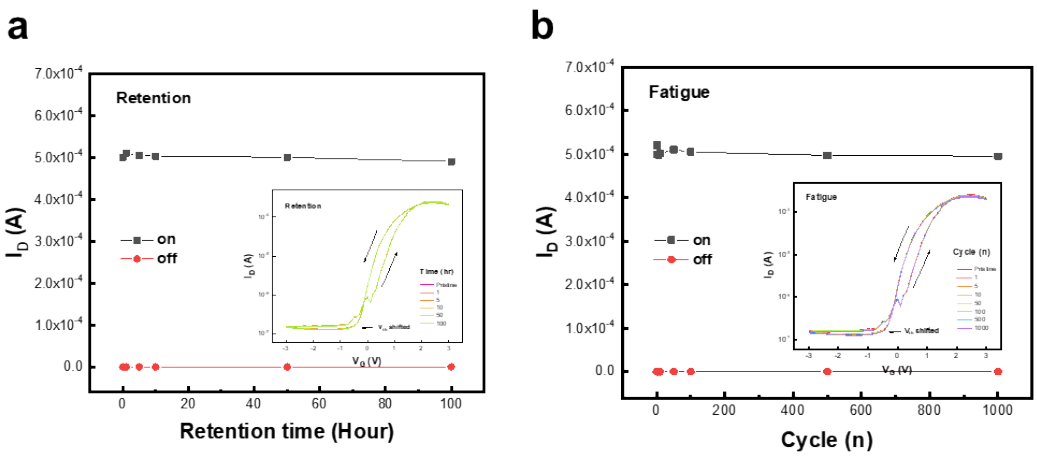

Figure. S8. The **a** retention and **b** endurance data of the FeFTT.

The FET with non-ferroelectric STO gate

A bottom-gate BOSe transistor based on the BOSe/STO/Nb:STO as shown in **Figure S9**a. The transfer characteristic has been investigated to check whether the influence of BOSe's ferroelectricity is critical. From the observation of **Figure S9**b, the typical hysteresis window is hardly observed in the I_d-V_g curves. Such a result further verifies the tiny influence of BOSe, and the effect of non-volatile modulation is contributed from the ferroelectric PZT layer.

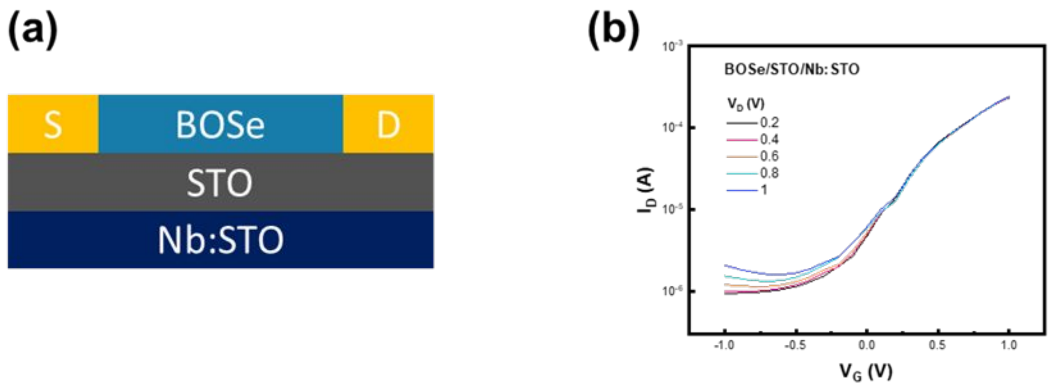

Figure S9. a. The schematic diagram of BOSe/STO/Nb:STO. b. The I_D-V_G curves of the synthesized structure with the STO gate.
